# White matter microstructure and network-connectivity in emerging adults with subclinical psychotic experiences

**DOI:** 10.1007/s11682-019-00129-0

**Published:** 2019-06-10

**Authors:** Stijn Michielse, Iris Lange, Jindra Bakker, Liesbet Goossens, Simone Verhagen, Marieke Wichers, Ritsaert Lieverse, Koen Schruers, Therese van Amelsvoort, Jim van Os, Machteld Marcelis

**Affiliations:** 1 Department of Psychiatry and Neuropsychology, School of Mental Health and Neuroscience, EURON, Maastricht University Medical Centre, PO Box 616, 6200 MD Maastricht, the Netherlands; 2 Department of Neuroscience, Center for Contextual Psychiatry, KU Leuven, Leuven, Belgium; 3 University Medical Center Groningen, Department of Psychiatry, Interdisciplinary Center Psychopathology and Emotion Regulation (ICPE), University of Groningen, Groningen, The Netherlands; 4 Faculty of Psychology, Center for Experimental and Learning Psychology, University of Leuven, Leuven, Belgium; 5 King’s Health Partners, Department of Psychosis Studies, Institute of Psychiatry, King’s College London, London, England; 6 Department of Psychiatry, Brain Center Rudolf Magnus, University Medical Center Utrecht, Utrecht, The Netherlands; 7 Institute for Mental Health Care Eindhoven (GGzE), Eindhoven, the Netherlands

**Keywords:** White matter, Emerging adults, Psychotic experiences, Network connectivity

## Abstract

Group comparisons of individuals with psychotic disorder and controls have shown alterations in white matter microstructure. Whether white matter microstructure and network connectivity is altered in adolescents with subclinical psychotic experiences (PE) at the lowest end of the psychosis severity spectrum is less clear. DWI scan were acquired in 48 individuals with PE and 43 healthy controls (HC). Traditional tensor-derived indices: Fractional Anisotropy, Axial Diffusivity, Mean Diffusivity and Radial Diffusivity, as well as network connectivity measures (global/local efficiency and clustering coefficient) were compared between the groups. Subclinical psychopathology was assessed with the Community Assessment of Psychic Experiences (CAPE) and Montgomery–Åsberg Depression Rating Scale (MADRS) questionnaires and, in order to capture momentary subclinical expression of psychosis, the Experience Sampling Method (ESM) questionnaires. Within the PE-group, interactions between subclinical (momentary) symptoms and brain regions in the model of tensor-derived indices and network connectivity measures were investigated in a hypothesis-generating fashion. Whole brain analyses showed no group differences in tensor-derived indices and network connectivity measures. In the PE-group, a higher positive symptom distress score was associated with both higher local efficiency and clustering coefficient in the right middle temporal pole. The findings indicate absence of microstructural white matter differences between emerging adults with subclinical PE and controls. In the PE-group, attenuated symptoms were positively associated with network efficiency/cohesion, which requires replication and may indicate network alterations in emerging mild psychopathology.

**Electronic supplementary material:**

The online version of this article (10.1007/s11682-019-00129-0) contains supplementary material, which is available to authorized users.

## Introduction

Over the last decades, white matter ‘integrity’ alterations have been frequently reported as a neural characteristic associated with psychotic disorder (Fornito et al. 2012; Friston 1999; Stephan et al. 2009). Decreased fractional anisotropy (FA, an index for white matter ‘integrity’) in fronto-temporal and fronto-limbic connections has also been described in help-seeking individuals considered at ultra-high risk for psychosis (Vijayakumar et al. 2016). The basis of water diffusion in cerebral white matter can be characterised by FA (reflecting both degree of myelination and coherence of fiber tracts), but also by other indicators such as mean diffusivity (MD; indicates free water movement in the white matter), axonal density (AXD; diffusion along the fibre axis indicating axonal packing) and radial diffusivity (RAD; diffusion perpendicular to the axis indicating myelin content) (Jones et al. 2013). Lower AXD and increased RAD have respectively been related to a lower number of axons and decreased myelin content (Peter J. Basser and Pierpaoli 2011). Previous research on patients with psychotic disorder suggests that AXD is unaffected (Kikinis et al. 2015; Reid et al. 2016; Scheel et al. 2013), whereas RAD and MD may be increased with respect to controls (Ardekani et al. 2011; Clark et al. 2011; Zeng et al. 2016). In addition, higher levels of positive psychotic symptoms have been associated with a decrease in FA (Lener et al. 2015; A. M. Michael et al. 2008b) and with altered RAD, AXD or MD (Andrew M. Michael et al. 2008a).

Another element in understanding structural connectivity and alterations therein is the network-based approach. This method is complimentary to the above described more traditional diffusion-weighted measures (DWI) and uses tractography as a basis to connect regional networks. As white matter alterations in psychotic disorder are spatially widespread (Ellison-Wright and Bullmore 2009), convergence can be achieved through network-based connectivity (Klauser et al. 2017). Indeed, studies have pointed towards alterations in white matter network connectivity as expressed by widespread fibre bundle disruption (Klauser et al. 2017), decreased global efficiency (Qifeng Wang et al. 2012a), fewer prefrontal hubs (brain regions facilitating integration) (M. Rubinov and Bullmore 2013; van den Heuvel and Fornito 2014) and decrease of frontal lobe nodes (van den Heuvel et al. 2010; Zhou et al. 2015). Additionally, decreased local efficiency (defined as how well information circulates over the network (Latora and Marchiori 2003)) has been described in frontal, temporal, (para)-limbic and putamen regions (Q. F. Wang et al. 2012b; Yan et al. 2015). Findings on the clustering coefficient (CC; measure of local cohesiveness/interconnectedness) are inconsistent, showing increased (Zhang et al. 2012), decreased (Li et al. 2018) and unchanged CC (Yeo et al. 2016). Furthermore, studies have reported negative associations between structural network efficiency on the one hand, and negative and positive symptoms in patients with psychotic disorder on the other (Skudlarski et al. 2010; Qifeng Wang et al. 2012a).

While knowledge has accrued regarding microstructural white matter alterations in psychotic disorder, little is known about AXD, RAD and MD in individuals with early stage, mild psychopathology, such as attenuated psychotic symptoms. Individuals with expression of attenuated psychotic symptoms are at increased risk for later stage emergence or persistence of psychopathology (van der Gaag et al. 2013). This group of individuals can be distinguished from clinical psychotic disorder based on the lower intensity and frequency of symptoms and associated distress of the attenuated symptoms (van Os et al. 2009). Attenuated psychotic symptoms have an estimated prevalence of 7.2% in the general population (DeRosse and Karlsgodt 2015), and a similar prevalence (6.9%) of attenuated psychotic symptoms has been described in adolescent populations (Fusar-Poli et al. 2013). The existing literature on structural white matter changes in individuals with subclinical psychotic experiences (PE) involves samples with diverse definitions and risks. One study found an association between increased psychosis-proneness and decreased FA in the frontal lobe in individuals with psychosis-linked personality traits (Grazioplene et al. 2016). Another study in young adults at familial risk for developing psychosis, reported decreased FA and increased RAD in association and projection fibers in the left hemisphere (Koivukangas et al. 2016). In contrast, increased FA and MD have been found in help-seeking individuals with so-called Ultra-High-Risk (UHR) status compared to controls (Vijayakumar et al. 2016). A one-year follow up study on individuals with an ‘at risk mental state’ (ARMS), found decreased FA and that only the individuals without transition to psychosis showed improvement of symptom levels associated with an increase in FA over the follow-up period (Katagiri et al. 2015).

Previous studies have also examined network-connectivity alterations in individuals with subclinical PE compared to controls. One study found lower betweenness centrality (the number of shortest paths passing through a region) in a sample with subclinical expression of psychosis, indicating a disruption of the backbone of the hub regions (van Dellen et al. 2016). Furthermore, help-seeking participants considered to have an ARMS showed reduced mean strength of the rich-club organisation (how well connected a network is), and decreased local efficiency in the right accumbens, while global efficiency was preserved (Schmidt et al. 2017). However, other studies showed increased local efficiency and decreased global efficiency (Choi et al. 2017), or reduced global efficiency and density with no alterations in mean clustering coefficient and betweenness (Drakesmith et al. 2015). Overall, network measures may be altered in individuals with PE, but the literature to date has not yielded a consistent pattern. Evidence for an association between attenuated symptoms and network-connectivity comes from a report on a negative association between negative symptoms and rich-club organisation in a help-seeking clinical high risk sample (Schmidt et al. 2017), but there is also a finding of absence of association between attenuated symptoms and network-connectivity measures in young adults at familial risk for psychotic disorder (Guusje Collin et al. 2017).

The current study combines network-based measures with traditional DWI measures in order to better understand structural white matter alterations in emerging adults with mild attenuated symptomatology at the lowest end of the psychosis severity spectrum who were not help-seeking (subclinical psychotic experiences and depressive symptoms, hereafter: PE-group). Based on the above described white matter alterations in psychotic disorder and the sparse findings in subclinical samples, it was hypothesized that the PE-group would display altered FA, AXD, RAD and MD with respect to controls (given the fact that the literature described both higher and lower microstructural white matter in PE). We also hypothesize lower global efficiency and local efficiency and a non-deviated clustering coefficient in the PE-group compared to controls. Lastly, in the PE-group, associations between symptoms and DWI white matter (network) alterations were explored. Attenuated symptoms were assessed cross-sectionally using questionnaires. In addition, we examined momentary measures of psychopathology using Experience Sampling technology, shedding light on symptoms dynamics in the continuous flow of daily life, which arguably may be closer to cerebral dynamics than the traditional static, period-based psychopathology measures (van Os et al. 2017). Since the literature is inconclusive with respect to regional DWI changes and associated attenuated symptoms, a region-based, complementary to the whole brain approach, was applied.

## Methods

### Participants

The study took place within the Smartscan project (Dutch Trial Register Number: NTR3808), comprising a sample of individuals between 16 and 25 years of age with subclinical PE either or not in combination with subclinical symptoms of depression (PE-group), a spider phobia sample (not included in the current paper) and a healthy control group, recruited in the area of Southern Limburg in the Netherlands. Recruitment was done via local advertisement, posters and presentations. The advertisement was aimed at individuals with mild psychopathology using statements people could identify with.

The inclusion criteria for the PE-group were based on the Community Assessment of Psychic Experiences (CAPE (Stefanis et al. 2002)) positive subscale frequency score ≥ 10 and/or positive subscale distress score ≥ 2. Participants with a Montgomery–Åsberg Depression Rating Scale (MADRS) (Montgomery and Asberg 1979) score ≥ 10 (McGorry et al. 2006) who met the subclinical PE inclusion criteria were also included. Participants were excluded if they had current psychological or psychiatric treatment and/or a significant need for care, i.e. they were non-help seeking. Inclusion criteria for the control group were based on MADRS <10, CAPE positive subscale frequency score < 10 and CAPE positive subscale distress <2. Individuals with a current and/or lifetime diagnosis on the Mini International Neuropsychiatric Interview (MINI) (Overbeek et al. 1999) and current and/or lifetime psychological or psychiatric treatment.

Exclusion criteria were: left-handedness, a history of neurological disorder (e.g. severe brain injury with unconsciousness, meningitis, migraine or epilepsy) and MRI contraindications (e.g. diabetes, claustrophobia, participants with inclusions of ferromagnetic material, and women with (suspected) pregnancy). Participants could take part in the Smartscan study if they fulfilled the in- and exclusion criteria.

The local medical ethics committee approved the study according to the declaration of Helsinki. All participants gave written informed consent in person and in addition a parent when younger than 18 years of age.

### Clinical measures

Lifetime cannabis and other drug use were assessed with the Composite International Diagnostic Interview (CIDI) section L (WHO 1990). Lifetime use was calculated by multiplying the number of weeks of use times the weekly frequency. Level of education was defined by completed level of education ranging from 0 (no education) to 7 (master degree).

The scores of the MADRS, CAPE positive, negative, depressive dimension as well as the CAPE total score on both the frequency and distress scales were calculated by summing the relevant items (Stefanis et al. 2002). To provide insight on the correlations between the individual subclinical symptoms, Spearman correlation coefficients between GAF, MADRS total score, CAPE positive symptom frequency/distress, negative symptom frequency/distress, depressive symptom frequency/distress and total symptom frequency/distress were calculated.

Momentary measures of subclinical PE in daily life were monitored using the Experience Sampling Method (ESM). The questions ‘I feel unreal’ and ‘I feel suspicious’ covered the psychosis dimension, based on previous work in this area (van Os et al. 2017; P. Delespaul 1995a). The item-score ranged from 1 (not at all) to 7 (very) on a Likert-scale. Individuals were asked to self-monitor their momentary mental state with the PsyMate (an electronic device with a touchscreen to record answers) during 15 days. The average over the items ‘I feel unreal’ and ‘I feel suspicious’ was calculated per individual as a composite momentary PE-score. At ten semi-random times during the day, the PsyMate signaled the participant with a beep, after which a short (5 min) questionnaire was completed within 15 min. Data were included if >30% of the completed beep questions were available, conform earlier work (PEAG. Delespaul 1995) (in the present study >45 out of 150 beeps). As a result, the number of individuals in the ESM analyses was 40 participants in each group (three participants in the HC-group and eight in the PE-group were excluded). This sample was used in the ESM analyses, while for all other analyses the entire sample was used.

### MRI acquisition

The MRI scans were acquired on a 3 T Siemens Magnetom Prisma system (Siemens, Erlangen, Germany) equipped with a 64-channel head/neck coil at Scannexus, Maastricht, The Netherlands. T1-weighted Magnetization Prepared Rapid Acquisition Gradient Echo (MPRAGE) whole brain images were acquired with a voxel size of 1.0 mm × 1.0 mm × 1.0 mm (repetition time (TR) = 2250 msec, echo time (TE) = 2.21 msec, flip angle = 9°, field of view (FOV) = 256 × 256, 192 sagittal orientated slices, GRAPPA = 2, no fat suppression, acquisition time (TA) = 5.05 min).

Whole brain structural Diffusion Weighted Imaging scans were acquired using an interleaved echo-planar-imaging sequence (field of view 200 × 200 mm^2^, TR 7300 ms, TE 49 ms, voxel size 2 × 2 × 2 mm^3^, b-value 1000 s/mm^2^, 72 slices, no overlap). 119 directions were recorded; 11 B0 volumes and 108 B-1000 volumes. Total acquisition time was 14m52s. Due to a scanner update a one scan (PE-group) was recorded with TR 7800 ms.

### Diffusion weighted imaging processing

Visual inspection of each dataset was performed and mean DWI signal intensity per volume in each dataset was calculated to check for artefacts and control data quality (see Supplementary Excel document, sheet 1). None of the values exceeded the threshold of three times the deviation from the mean. In addition, the mean and standard deviation of DWI signal intensity between HC and PE groups were statistically compared with a regression test for both B0 and B1000 volumes. In addition, Pearson correlations between mean DWI signal and symptoms were calculated over the entire sample. After conversion of raw DICOM images to NIfTI format (Rorden and Brett 2000), the DWI data were converted from NIfTI standard to .mat standard using ExploreDTI (Leemans and Jones 2009) in a MatLab (The MathWorks, Inc., Natick, Massachusetts, United States) programming environment. Motion and eddy-current induced geometrical distortions were corrected by realigning the diffusion images to the B0 images incorporating B-matrix rotation (Leemans and Jones 2009) and coregistered to the individual’s anatomical data to correct for echo-planar imaging (EPI) distortions as implemented in Elastix (Irfanoglu et al. 2012; Klein et al. 2010). The diffusion tensor metrics were calculated using the Robust Estimation of Tensors by Outlier Rejection (RESTORE) method (Chang et al. 2005). FA was calculated via this formula: $\frac{1}{2}\frac{\sqrt{\left({\left({\lambda}_1-{\lambda}_2\right)}^2+{\left({\lambda}_2-{\lambda}_3\right)}^2+{\left({\lambda}_3-{\lambda}_1\right)}^2\right)}}{\sqrt{{\lambda_1}^2+{\lambda_2}^2+{\lambda_3}^2}}$ in which λ_1_ is the primary, λ_2_ the secondary and λ_3_ the tertiary eigenvector, AXD is the λ_1_ is the primary eigenvector and RAD the average of the λ_2_ and λ _3_ eigenvectors. FA maps were calculated with a threshold of >0.2. White matter tracts were reconstructed at 1mm^3^ resolution using deterministic fiber tractography, for each individual dataset (P. J. Basser et al. 2000). Deterministic tractography is based on the underlying anatomical structures as described earlier using Elastix and the RESTORE algorithm. Individual MD, AXD and RAD maps were exported from ExploreDTI to be used in the next steps.

### Tract-based analysis

Tract-based spatial statistics (TBSS) in FSL 5.0.9 (FMRIB Analysis Group, Oxford, UK) was effectuated for further processing of the DWI data. Nonlinear registration aligned all FA volumes to 1 × 1 × 1 mm standard FMRIB58_FA space. The standard FMRIB58_FA consists of a template derived from high-resolution images of 58 participants (males and females between 20 and 50 years of age) (Smith et al. 2006). A mean FA skeleton based on the whole sample was generated. This skeleton follows the major white matter tracts in each individual participant (normalized in MNI152 space) and provides a way to examine group differences. The FA threshold was set at 0.2 after visual inspection of the FA skeleton in order to include major white matter tracts while removing small peripheral tracts that would cause excess inter-participant variability. A voxel-wise statistical analysis was performed based on the mean FA skeleton using a general linear model applying permutation testing using FSL’s randomise (v2.9) (Winkler et al. 2014). Based on the FA processing, the mean MD, AXD and RAD were extracted.

### Group differences in tensor-derived indices

#### Whole brain analyses

Statistical evaluation of group differences in mean FA, AXD, RAD and MD was performed in two directions: PE-group > controls and PE-group < controls. The a priori hypothesized confounding variables age, sex, level of education, lifetime cannabis use and other lifetime drug use (Cookey et al. 2014) were added to this and other statistical models described below. A total of 5000 permutations were used. A threshold-free cluster enhancement (TFCE) threshold of *p* > 0.05 was applied to all statistical tests in TBSS (Salimi-Khorshidi et al. 2011).

Furthermore, in the PE group, whole-brain-symptom correlational analyses were conducted in TBSS with the same tensor-derived parameters (FA, AXD, RAD and MD).

#### ROI analyses

In order to investigate group differences in 38 white matter regions based on the Johns Hopkins University International Consortium for Brain Mapping (JHU ICBM)-DTI-81 atlas labels (Mori et al. 2008) the mean FA, AXD, RAD and MD per region of interest (ROI) were computed per individual resulting in a hierarchical dataset (38 assessments per DWI measure clustered within participants). The ROI’s were based on the skeletonized white matter tracts generated by TBSS and were normalized to MNI152 space conform previous papers (Domen et al. 2013; Michielse et al. 2017). This data was export and statistically analyzed in (R Development Core Team 2008). The interactions between group and ROI (dummy coded 1 till 38) in the models of FA, AXD, RAD and MD were investigated using a multilevel random effects model. Group status was coded ‘1’ for PE-group and ‘0’ for HC-group. The number of voxels was added to the model as an analytic weight to control for ROI size (i.e., the error variance for a particular observation was inversely weighted by the number of voxels within the corresponding region). In case of a significant interaction between group and ROI (*p* < 0.05), the association between group and DWI measure was tested per ROI. Bonferroni correction was applied to control the type I error rate, since group differences in 38 regions were tested.

### Group differences in network parameters

Parcellation of the whole brain fiber tracts was done using the standard automated anatomical atlas labeling (AAL, (Tzourio-Mazoyer et al. 2002)). The fiber tracts range from one region to the other, providing a cortical coverage, and they end or start in each of the specified AAL regions. This procedure provides 90 (sub)-cortical brain ROIs, each representing a node in the white matter network. The reconstructed white matter tracts were represented as edges between each pair of nodes. The AAL atlas provides proper cortical coverage and is widely used in both structural and functional connectivity analysis. With the use of the individual brain networks, connectivity measures were computed in order to quantify the network architecture using the Brain Connectivity Toolbox (Mikail Rubinov and Sporns 2010). The connectivity measures derived from the AAL tractography were global/local efficiency and clustering coefficient. Efficiency quantifies the exchange of information on a global or local nodal level. Clustering coefficient indicates to what extent nodes in a network tend to cluster together. Network connectivity was described in terms of local efficiency and clustering coefficient (Mikail Rubinov and Sporns 2010) based on the pass criteria (i.e., where the tract passes through) per ROI node. All three connectivity network measures (global efficiency, local efficiency and clustering coefficient) were computed per individual, exported and statistically analyzed in (R Development Core Team 2008).

#### Global efficiency

Group differences in global efficiency were assessed with a multiple linear regression model (wide format).

#### Local efficiency and clustering coefficient

The interaction between group and ROI in the models of local efficiency and clustering coefficient was investigated using a multilevel random effects model, including a random effect for participant (long format). The number of voxels was added to the model as an analytic weight to control for ROI size (i.e., the error variance for a particular observation was inversely weighted by the number of voxels within the corresponding region). In case of a significant interaction between group and ROI (*p* < 0.05), the association between group and DWI measure was tested per ROI. Bonferroni correction was applied to control the type I error rate, since group differences in 90 regions were tested.

### Associations between attenuated symptoms and tensor-derived indices

Within the PE-group, the interactions between the CAPE scores and ROI in the models of DWI measures FA, AXD, RAD and MD were analyzed in multilevel random regression models with a random intercept for participants. The same model was used for all four CAPE dimensions (frequency/distress scores), the MADRS total score and the ESM momentary composite PE-score. In case of a significant interaction between symptom and ROI (at a conservative level of *p* < 0.01 given multiple tests with symptoms scores), the association between symptom score and DWI measure was tested per ROI. Bonferroni correction was applied to control the type I error rate, since a total of 38 tests were conducted at ROI level. The analyses with the CAPE and ESM PE-scores were repeated controlling for the total MADRS score.

### Associations between attenuated symptoms and network connectivity parameters

#### Global efficiency

Within the PE-group, the association between CAPE score (independent variable) and connectivity measure (global efficiency; dependent variable) was investigated using a multiple linear regression model. The same model was used for all four CAPE dimensions (frequency/distress), the MADRS total score and the ESM composite PE-score.

#### Local efficiency and clustering coefficient

Within the PE-group, the interaction between CAPE score and ROI in the models of local efficiency and clustering coefficient were analyzed with multilevel random regression. The same model was applied for all four CAPE dimensions (frequency/distress scores), MADRS total score and ESM momentary composite PE-score. In case of a significant interaction between symptom and ROI (at a conservative level of *p* < 0.01 given multiple tests with symptoms scores), the association between symptom score and DWI measure was tested per ROI. Bonferroni correction was applied to control the type I error rate, since a total of 90 hypotheses were tested. These analyses with the CAPE and ESM PE-score were repeated controlling for the total MADRS score.

## Results

### Participant characteristics

For the current study, a total of 48 participants were included in the PE-group and 43 in the healthy control (HC)-group. Of note, a total of 199 participants was included in the initial sample of the Smartscan project. For the current analyses, participants with spider phobia (*n* = 60), incomplete ESM assessment (*n* = 12 and DWI artefacts (*n* = 2) were excluded from this sample. In addition, several participants decided to withdraw after inclusion (*n* = 27), while some had unforeseen MRI contra-indications (*n* = 7).

Groups were comparable with regard to age, sex, educational level, cannabis use and lifetime drug use and differed in CAPE symptom scores, MADRS score and GAF score as shown in Table 1. In the PE-group there were 11 participants included based on only the CAPE score, while the remaining 37 also had subclinical depressive experiences (MADRS ≥10). Individuals in the PE-group reported higher levels of momentary ESM composite PE-score in daily life compared to the HC-group (Table 1).

**Table 1:** Participant characteristics

	HC (*n* = 43)	PE (*n* = 48)
Age (mean (sd))	21.81 (1.69)	21.31 (2.48)
Sex female (%)	35 (81.4)	37 (77.1)
Educational level:		
Lower general education (%)	0 (0.0)	1 (2.1)
Vocational education (%)	1 (2.3)	2 (4.2)
High school (%)	2 (4.7)	5 (10.4)
Higher education (Bachelor) (%)	29 (67.4)	37 (77.1)
University (Master) (%)	11 (25.6)	3 (6.2)
CAPE positive frequency (mean (sd))	0.84 (1.38)	4.71 (2.99)
CAPE positive distress (mean (sd))	0.16 (0.37)	4.33 (2.90)
CAPE negative frequency (mean (sd))	3.93 (2.78)	14.14 (6.30)
CAPE negative distress (mean (sd))	2.14 (2.14)	14.50 (8.14)
CAPE depressive frequency (mean (sd))	1.88 (1.55)	9.08 (4.50)
CAPE depressive distress (mean (sd))	1.44 (1.86)	10.35 (5.23)
CAPE total frequency (mean (sd))	6.65 (4.69)	27.92 (11.89)
CAPE total distress (mean (sd))	3.74 (3.76)	29.21 (14.11)
MADRS score (mean (sd))	1.35 (1.81)	13.58 (7.16)
GAF score (mean (sd))	86.81 (6.34)	62.17 (11.17)
Cannabis lifetime (mean (sd))	3.05 (9.93)	8.15 (20.25)
Other drugs lifetime (mean (sd))	0.33 (1.49)	11.06 (50.54)
PE-score (mean (sd))*	1.10 (0.17)	1.66 (0.86)

Means, standard deviations and frequencies are provided per group. HC-group: Healthy Control group; PE-group: Subclinical Psychotic Experiences group; CAPE: community assessment of psychic experiences; MADRS: Montgomery–Åsberg Depression Rating Scale; GAF: Global Assessment of Functioning. The total lifetime cannabis and other drug use were calculated by multiplying the frequency per week times the number of weeks of use. * ESM data were missing for three participants in the HC-group and eight in the PE-group.

The symptom correlation analyses showed positive relations between CAPE and MADRS symptom scores and especially CAPE frequency and distress were strongly correlated. The GAF score had a negative correlation with both CAPE and MADRS symptom scores (Supplementary Table 1).

The mean DWI signal intensity was equal between the two groups (B0; Mean (standard deviation) PE-group 283(31), HC-group 282(36), PE-group vs. HC-group: B = 1.05, *p* = 0.98, B1000; Mean PE-group 109(11), HC-group 109(12), PE-group vs. HC-group: B = 0.09, *p* = 0.99). The DWI signal did not correlate with the symptom scores in the entire sample (Supplementary Table 2).

In 13 participants pertaining to the PE-group, psychotropic medications had been used in the past: selective serotonin reuptake inhibitors (2 participants), methylphenidate (4 participants), melatonine (4 participants), risperidone (1 participant), minor tranquilizers / benzodiazepines (5 individuals) and bupropion (1 participant). The number of individuals currently using psychotropic medication was 2 (both in the PE-group). These individuals used a minor tranquilizer (Valerian) and quetiapine (25 mg at night) when needed. None of the participants were ever admitted to hospital for a mental disorder.

### Group differences in tensor-derived indices

#### Whole brain analyses and ROI analyses

Whole brain TBSS analyses did not show significant differences between the groups for any of the DWI measures. Additionally, no significant interaction between group and ROI in the model of FA (χ^2^ = 34.64, *p* = 0.53), AXD (χ^2^ = 27.60, *p* = 0.84), RAD (χ^2^ = 42.96, *p* = 0.20) and MD (χ^2^ = 40.22, *p* = 0.29) were found (Table 2).

**Table 2:** Group differences in tensor-derived indices and network connectivity measures

	PE-group Mean (sd)	HC-group Mean (sd)	B	χ^2^	*p* value
Fractional Anisotropy	0.58 (0.084)	0.58 (0.083)	n/a	34.64	0.53
Axial Diffusivity	0.0014 (0.00018)	0.0014 (0.00017)	n/a	27.60	0.84
Radial Diffusivity	0.00048 (0.00079)	0.00048 (0.00076)	n/a	42.96	0.20
Mean Diffusivity	0.00077 (0.00074)	0.00077 (0.00072)	n/a	40.22	0.29
Global efficiency	0.72 (0.017)	0.72 (0.017)	0.0045	n/a	0.22
Local efficiency	0.85 (0.048)	0.85 (0.049)	n/a	62.55	0.98
Clustering coefficient	0.35 (0.049)	0.35 (0.050)	n/a	67.46	0.96

Means and standard deviations are provided per group. χ^2^, estimates and *p* values are derived from multilevel random regression analyses. B is the coefficient derived from the statistical model reflecting the estimated difference in global efficiency between the groups. n/a = not applicable.

PE-group = Subclinical Psychotic Experiences group, HC-group = Healthy Control group.

### Group differences in network connectivity measures

#### Global efficiency

Global efficiency (B = 0.0045, *p* = 0.22) was not statistically different between groups (Table 2).

#### Local efficiency and clustering coefficient

The interaction between group and ROI was not significant in the model of local efficiency (χ^2^ = 62.55, *p* = 0.98) or in the model of clustering coefficient (χ^2^ = 67.46, *p* = 0.96) (Table 2).

### Associations between subclinical symptoms and tensor-derived indices in the PE-group

#### CAPE and MADRS scores

There were no significant interactions between any of the CAPE symptom score dimensions and ROI in the models of FA, AXD, RAD and MD (Table 3). Similarly, there was no significant interaction between MADRS total score and ROI in the models of FA, AXD, RAD and MD (Table 3). The findings did not change when the CAPE and ESM models were controlled for the total MADRS score (Supplementary Table 3).

**Table 3:** Interactions between attenuated symptoms and ROI in the models of tensor-derived indices within the PE group

	FA		AXD		RAD		MD	
	χ^2^	*p* value	χ^2^	*p* value	χ^2^	*p* value	χ^2^	*p* value
CAPE positive								
* frequency score*	25.69	0.92	26.52	0.90	23.32	0.96	16.00	0.99
* distress score*	38.69	0.39	37.62	0.44	31.93	0.71	22.23	0.98
CAPE negative								
* frequency score*	24.77	0.94	20.17	0.99	22.79	0.97	26.54	0.92
* distress score*	29.49	0.81	22.27	0.97	41.20	0.29	51.64	0.07
CAPE depressive								
* frequency score*	30.77	0.76	25.06	0.93	35.03	0.56	43.10	0.26
* distress score*	31.36	0.73	32.11	0.70	38.70	0.39	56.52	0.03
CAPE total								
* frequency score*	26.60	0.90	21.33	0.98	24.37	0.95	25.14	0.95
* distress score*	30.73	0.76	23.21	0.96	40.82	0.31	50.28	0.09
MADRS								
* total score*	28.42	0.84	41.94	0.27	22.99	0.97	43.09	0.26
Daily life ESM								
* PE-score*	21.28	0.98	19.85	0.99	17.92	0.99	14.23	0.99

*FA* Fractional Anisotropy, *AXD* Axial Diffusivity, *RAD* Radial Diffusivity and *MD* Mean Diffusivity, *CAPE* Community Assessment of Psychic Experiences, *MADRS* Montgomery–Åsberg Depression Rating Scale, *ESM* Experience Sampling Method

χ^2^, estimates and *p* values are derived from multilevel random regression analyses

Results from the whole-brain TBSS correlation analyses showed no significant voxels.

#### ESM momentary subclinical psychosis scores

There were no significant interactions between ROI and PE-score in any of the DWI white matter models (Table 3).

### Associations between subclinical symptoms and network connectivity parameters in the PE-group

#### CAPE and MADRS scores in association with global efficiency

There was no significant association between any of the CAPE or MADRS scores and global efficiency (Table 4).

**Table 4:** Association between subclinical symptoms and network connectivity measures within the PE group

	Global efficiency		Local efficiency		Clustering coefficient	
	Estimate	*p* value	χ^2^	*p* value	χ^2^	*p* value
CAPE positive						
* frequency score*	0.000038	0.97	149.91	<0.0001	127.09	0.006
* distress score*	0.00024	0.80	138.26	0.0006	128.67	0.005
CAPE negative						
* frequency score*	0.00040	0.33	119.10	0.02	107.87	0.10
* distress score*	0.00019	0.55	97.17	0.28	87.31	0.56
CAPE depressive						
* frequency score*	0.00012	0.84	93.04	0.39	80.50	0.75
* distress score*	−0.00021	0.97	88.24	0.53	84.13	0.65
CAPE total						
* frequency score*	0.00014	0.54	118.76	0.02	101.29	0.20
* distress score*	0.000066	0.61	102.67	0.17	91.02	0.45
MADRS						
* total score*	−0.00016	0.64	66.30	0.97	72.98	0.90
Daily life ESM						
* PE-score*	−0.0084	0.97	77.90	0.81	72.49	0.91

*MADRS* Montgomery–Åsberg Depression Rating Scale, *ESM* Experience Sampling Method

χ^2^, estimates and *p*-values are derived from multilevel random regression analyses (local efficiency and clustering coefficient) and regression for global efficiency

#### ESM momentary subclinical psychosis scores in association with global efficiency

The daily life composite ESM item PE-score showed no significant association with global efficiency (Table 4).

#### CAPE and MADRS scores in association with nodal network connectivity

The positive symptom frequency score of the CAPE showed a significant interaction with ROI in the model of local efficiency (χ^2^ = 149.91, *p* < 0.0001) and clustering coefficient (χ^2^ = 127.09, *p* = 0.006) (Table 4). After correction for multiple testing, no significant interactions remained (Supplementary Tables 4 and 5).

**Fig. 1:**
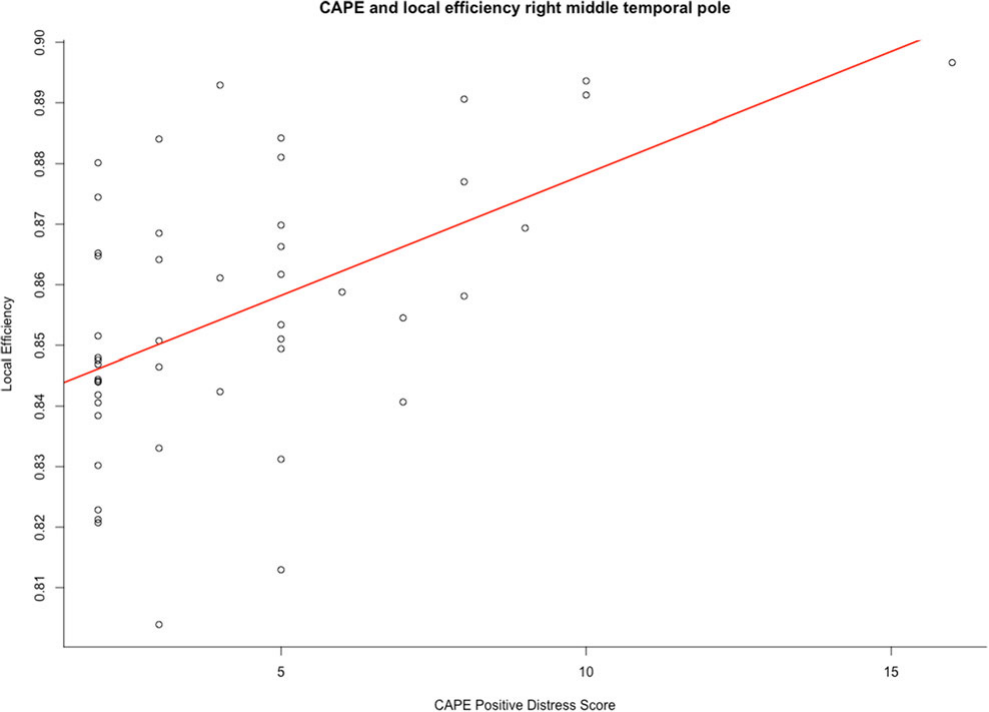
The significant association (B = 0.0040, *p* = 0.01) between local efficiency and CAPE positive distress score in the PE-group. CAPE; Community Assessment of Psychic Experiences

**Fig. 2:**
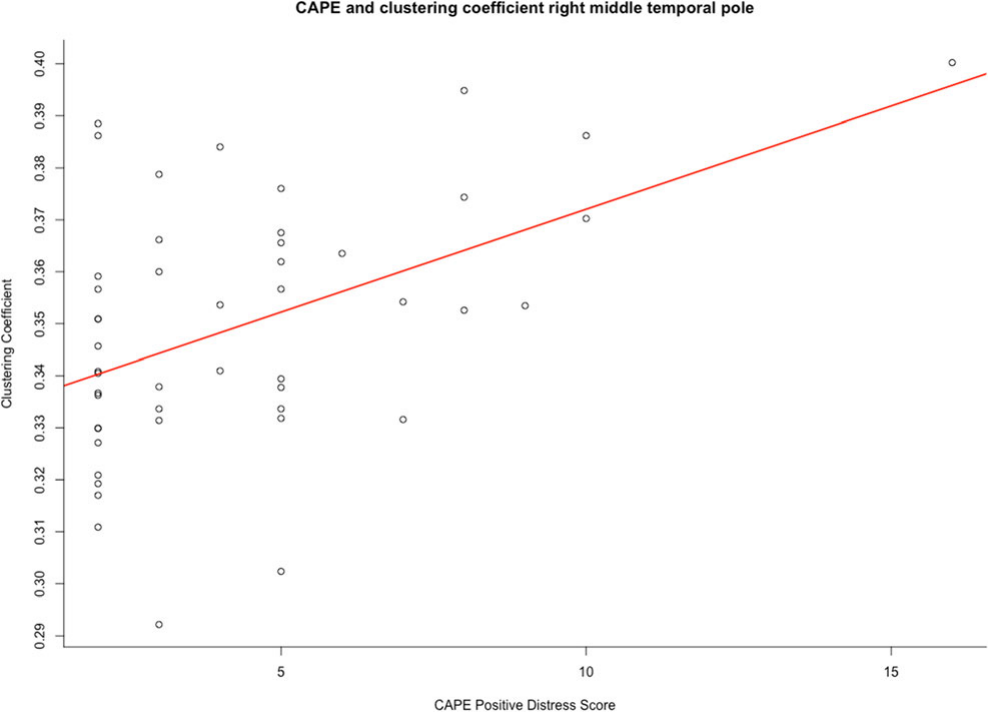
The significant association (B = 0.0039, *p* = 0.05) between clustering coefficient and CAPE positive distress score in the PE-group. CAPE; Community Assessment of Psychic Experiences

The positive symptom distress score of the CAPE showed a significant interaction with ROI in the model of local efficiency (χ^2^ = 138.26, *p* = 0.0006) and clustering coefficient (χ^2^ = 128.67, *p* = 0.005) (Table 4). Stratified analyses showed a significant increase in both local efficiency (b = 0.0040, *p* = 0.01, Fig. 1) and clustering coefficient (b = 0.0039, *p* = 0.05, Fig. 2) in the right middle temporal pole (MTP) with increasing levels of distress from positive symptoms, after Bonferroni correction (Supplementary Tables 6 and 7).

There were no significant interactions between ROI and the CAPE depressive symptom, negative symptom and total scores or the MADRS total score in the models of local efficiency and clustering coefficient (Table 4). The findings did not change when the CAPE and ESM models were controlled for the total MADRS score (Supplementary Table 8).

#### ESM momentary subclinical psychosis scores in association with nodal network connectivity

The ESM composite PE item showed no significant interaction with ROI in the models of local efficiency and clustering coefficient (Table 4).

## Discussion

This study showed that microstructural white matter measures and structural network connectivity parameters were not different between non-help seeking young individuals with subclinical PE and controls. While attenuated subclinical symptoms were not associated with white matter structural alterations, there was some evidence for an association between symptoms and network connectivity in the PE group. Explorative, hypothesis-generating analyses in this group showed positive associations between positive symptom distress scores and local efficiency and clustering coefficient in the right MTP.

### Structural disconnectivity in individuals with subclinical psychotic experiences

The findings showed that non-help seeking individuals with subclinical PE were not different from controls with regard to any of the tensor-derived indices. This is in contrast with previously described decreased FA and increased MD in help-seeking Ultra-High-Risk (UHR) samples (see meta-analysis based on 12 studies (Vijayakumar et al. 2016)), though in line with another UHR-study reporting absence of microstructural white matter differences in young adults with respect to controls (Koivukangas et al. 2015). Thus, the results suggest that non-help seeking individuals with subclinical PE have preserved white matter microstructure, in contrast to individuals with psychotic disorder (Ellison-Wright and Bullmore 2009), or to help-seeking individuals at high risk for psychotic disorder (Vijayakumar et al. 2016), who, at group level, show reduced white matter ‘integrity’. Compared to UHR studies, which include individuals who already are help-seeking in the context of mostly an existing affective disorder or a substance use disorder with a certain degree of psychosis admixture (van Os and Guloksuz 2017), the current study included individuals who were not help-seeking and likely had lower levels of psychopathology and psychosis admixture. There is also variation in age of the samples that have been studied (with the individuals of the current study being relatively young), implying differential stages of white matter maturation. To date, research on individuals at the attenuated, subclinical end of psychopathology remains sparse, though information on (early) phenomenological and biological differentiation is important to understand variation and alteration during development. In addition, it is informative with respect to efforts on clinical and biological staging and profiling (Koutsouleris et al. 2015).

### Structural network connectivity in individuals with subclinical psychotic experiences

No differences were found in the network-connectivity measures between the PE-group and controls. It was hypothesized that the frontal brain regions would show signs of disconnectivity in the fronto-occipital and fronto-temporal white matter, based on previous evidence in psychotic disorder (Klauser et al. 2017; M. Rubinov and Bullmore 2013; van den Heuvel and Fornito 2014; Qifeng Wang et al. 2012a). The literature on white matter network alterations in individuals with attenuated psychotic symptoms is rather limited and inconclusive, also due to different techniques that are used. Some studies have described alterations in rich-club organization (38, 39) and reduced local efficiency (Choi et al. 2017; Zhao et al. 2017; Schmidt et al. 2017) of the structural brain network in help-seeking individuals at high risk for psychotic disorder, which is likely different from the current, non-help seeking population. Moreover, it is challenging to compare studies, since high-risk sampling strategy, high-risk criteria, age groups, severity of symptomatology and network measures often differ between studies. The findings of the current study nevertheless suggest that network connectivity may be preserved in individuals with subclinical PE.

### Associations between symptoms and tensor-derived indices in individuals with psychotic experiences

In the current study, no associations were found between CAPE and MADRS symptom scores and DWI measures. This can be due to the sampling frame aimed at the lower end of psychosis severity spectrum. A previous cross-sectional study has pointed to positive associations between positive schizotypy symptoms and FA in a sample with nonclinical psychosis-linked personality traits (Grazioplene et al. 2016), while a longitudinal study in a help-seeking at-risk sample showed that improvement in positive symptoms was related to increased FA (Katagiri et al. 2015). Both studies were at higher levels of the psychosis severity spectrum, which may explain the difference.

Despite the fact that daily life assessment using ESM can be a more valid way of assessing mild symptoms as compared to filling in a questionnaire, since recall bias is reduced and questions are answered in the moment, and may be closer to brain dynamics in daily life, there was no evidence for associations between ESM subclinical PE and the DWI measures.

### Associations between symptoms and network measures in individuals with subclinical psychotic experiences

Explorative analyses on the associations between symptoms and network measures were conducted within the PE-group. There was a positive association between the CAPE positive symptom distress scores and local efficiency and clustering coefficient in the right MTP. While the association between symptom distress and structural network connectivity has not been described in individuals with (or at risk for) psychotic disorder, distress can be the difference between help-seeking and clinical relevance of psychotic experiences (Cohen and Davis 3rd 2009). Replication is required to assess whether network efficiency and cohesion within the right MTP may be an early sign of symptoms related to emerging psychosis.

Furthermore, no association between the frequency/distress of negative symptoms and local efficiency was found. Research on subclinical negative symptoms is limited, let alone in association with DWI network alterations. Two studies in individuals at high-risk for psychosis reported a negative association between subclinical negative symptoms and rich-club organization (Schmidt et al. 2017; Li et al. 2018), while another study reported no associations between network connectivity and subclinical negative symptoms (Zhao et al. 2017). In psychotic disorder, absence of an association between negative symptoms and network-based measures (Levitt et al. 2017), as well as a negative association between network connectivity and negative symptoms has been described (Qifeng Wang et al. 2012a). As emerging psychosis is often preceded by (subclinical) negative symptoms (Piskulic et al. 2012; Yung and McGorry 1996), and negative symptoms impact on daily life functioning (Blair et al. 2018), it is important to understand the association with cerebral network alterations in order to identify and improve ways of early detection and intervention.

The current study showed no significant associations between depressive symptoms (measured with CAPE and MADRS) and network connectivity measures. Research on the relation between depressive symptomatology and brain network connectivity is limited to several small studies and suggests that, in siblings of patients with psychotic disorder, rich-club connectivity was not associated with depressive symptom severity (G. Collin et al. 2014), while one study with an ARMS population, showed a correlation between rich-club disorganization and depressive symptom severity (Schmidt et al. 2017). Thus, based on sparse previous literature and findings from the current study, it has yet to be determined whether variations in brain network configuration co-occur with variation in depressive symptoms in individuals with subclinical PE.

Additionally to the use of more traditional questionnaires, we explored whether change in momentary PE measurement varied with brain network connectivity measures, which did not provide significant results. Since it was the first time that this research question was tested, no hypotheses were stated. It is thus clear that the current explorative and hypothesis-generating findings warrant further investigation.

### Methodological considerations

While the study was carefully designed, some considerations need to be taken into account. First, the PE-group had mild symptom levels, as inclusion was based on a CAPE positive distress score of ≥2, and help-seeking was excluded. Thus, the sampling frame targeted a group at the lowest level of the severity spectrum, below the high-risk sampling frame of help-seeking individuals with affective or substance use disorder and a degree of psychosis admixture. Whether the absence of differences between the groups reflects a true finding needs to be ascertained in future studies at the lower end of the psychosis severity spectrum. Second, the sample size did not allow for specific sensitivity analyses in subgroups with for example higher or lower symptom levels. It can be questioned whether this would provide more information against the dimensional approach. Overall the sample size was restricted and this should be taken into account in the interpretation of the results. Third, the association between attenuated symptoms and DWI measures was investigated only in the mild psychopathology group, as controls would have insufficient variance in symptomatology. Therefore, it is not known whether similar associations exist may in controls and it cannot be claimed that the findings are specific for the group under investigation.

The tensor estimation model had information on 119 directions to reconstruct the diffusion tensor in 2mm^3^ resolution. The RESTORE algorithm (P. J. Basser et al. 2000) is a widely used method for reducing the impact of outliers on the data, but novel techniques such as HARDI (high angular resolution diffusion imaging) or CHARMED (composite hindered and restricted model of diffusion) might improve the estimation (Jones et al. 2013). Lastly, the TBSS method might have been too crude to detect minor white matter changes. Since small white matter tracts were excluded in the processing, slight but relevant details may have been lost in the procedure.

## Conclusion

This study demonstrated absence of differences between individuals at the lowest end of the psychosis severity spectrum and controls with respect to FA, AXD, RAD and MD measures. Similarly, there were no network-based connectivity differences between the groups. In explorative analyses within the PE-group, some attenuated symptom measures were positively associated with network efficiency/cohesion.

## Electronic supplementary material

ESM 1 (XLSX 197 kb)

ESM 2 (DOCX 60 kb)
