# Supplementary material for: White matter microstructure and network-connectivity in emerging adults with subclinical psychotic experiences
Source: Brain Imaging Behav. 2019 Jun 10;14(5):1876–88. doi: 10.1007/s11682-019-00129-0 (PMC7572337; doi:10.1007/s11682-019-00129-0)
Supplement: Supplementary file 2 — (DOCX 60 kb) [file 11682_2019_129_MOESM2_ESM.docx]

Table 1. Spearman correlation matrix of subclinical symptoms measures.

|  | CAPE positive frequency | CAPE positive distress | CAPE depressive frequency | CAPE depressive distress | CAPE negative frequency | CAPE negative distress | CAPE total frequency | CAPE total distress | MADRS total score | GAF |
| --- | --- | --- | --- | --- | --- | --- | --- | --- | --- | --- |
| CAPE positive frequency | 1.00 |  |  |  |  |  |  |  |  |  |
| CAPE positive distress | 0.87 | 1.00 |  |  |  |  |  |  |  |  |
| CAPE depressive frequency | 0.74 | 0.84 | 1.00 |  |  |  |  |  |  |  |
| CAPE depressive distress | 0.74 | 0.83 | 0.95 | 1.00 |  |  |  |  |  |  |
| CAPE negative frequency | 0.79 | 0.79 | 0.84 | 0.79 | 1.00 |  |  |  |  |  |
| CAPE negative distress | 0.75 | 0.81 | 0.87 | 0.88 | 0.92 | 1.00 |  |  |  |  |
| CAPE total frequency | 0.87 | 0.87 | 0.93 | 0.89 | 0.97 | 0.93 | 1.00 |  |  |  |
| CAPE total distress | 0.79 | 0.88 | 0.93 | 0.96 | 0.89 | 0.97 | 0.94 | 1.00 |  |  |
| MADRS total score | 0.67 | 0.66 | 0.78 | 0.79 | 0.71 | 0.77 | 0.77 | 0.80 | 1.00 |  |
| GAF | -0.62 | -0.78 | -0.79 | -0.77 | -0.74 | -0.74 | -0.74 | -0.77 | -0.77 | 1.00 |

CAPE: Community Assessment of Psychic Experiences; MADRS: Montgomery–Åsberg Depression Rating Scale; GAF: Global Assessment of Functioning.

Table 2. Pearson correlations between mean DWI signal and symptoms in the entire sample.

|  | B0 | B1000 |
| --- | --- | --- |
| CAPE positive frequency | 0.12 | 0.04 |
| CAPE positive distress | 0.08 | 0.03 |
| CAPE depressive frequency | -0.05 | -0.13 |
| CAPE depressive distress | -0.06 | -0.12 |
| CAPE negative frequency | 0.03 | -0.05 |
| CAPE negative distress | -0.0003 | -0.07 |
| CAPE total frequency | 0.02 | -0.06 |
| CAPE total distress | -0.008 | -0.08 |
| MADRS total score | -0.01 | -0.08 |

CAPE: Community Assessment of Psychic Experiences; MADRS: Montgomery–Åsberg Depression Rating Scale

Table 3. Interactions between attenuated symptoms and ROI in the models of tensor derived indices within the PE group. Analyses are corrected for total MADRS score.

|  | FA | | AXD | | RAD | | MD | |
| --- | --- | --- | --- | --- | --- | --- | --- | --- |
|  | χ^2^ | p-value | χ^2^ | p-value | χ^2^ | p-value | χ^2^ | p-value |
| CAPE positive frequency score | 26.95 | 0.89 | 28.91 | 0.83 | 23.62 | 0.96 | 15.58 | 0.99 |
| CAPE positive distress score | 39.32 | 0.37 | 39.42 | 0.36 | 32.03 | 0.70 | 21.71 | 0.98 |
| CAPE negative frequency score | 25.81 | 0.92 | 19.95 | 0.99 | 23.29 | 0.96 | 25.67 | 0.92 |
| CAPE negative distress score | 31.19 | 0.74 | 22.25 | 0.97 | 41.97 | 0.26 | 50.11 | 0.07 |
| CAPE depressive frequency score | 33.97 | 0.61 | 23.84 | 0.95 | 37.20 | 0.46 | 42.39 | 0.25 |
| CAPE depressive distress score | 34.98 | 0.56 | 31.06 | 0.74 | 40.92 | 0.30 | 55.40 | 0.03 |
| CAPE total frequency score | 29.31 | 0.81 | 21.45 | 0.98 | 25.76 | 0.92 | 24.40 | 0.94 |
| CAPE total distress score | 33.62 | 0.63 | 23.38 | 0.96 | 42.20 | 0.26 | 48.85 | 0.09 |
| Daily life ESM PE-score | 21.37 | 0.98 | 14.28 | 0.99 | 17.90 | 0.99 | 14.22 | 0.99 |

FA; Fractional Anisotropy, AXD; Axial Diffusivity, RAD; Radial Diffusivity and MD; Mean Diffusivity. CAPE; Community Assessment of Psychic Experiences, MADRS; Montgomery–Åsberg Depression Rating Scale, ESM; Experience Sampling Method.

χ^2^, estimates and p-values are derived from multilevel random regression analyses.

Table 4. CAPE positive symptom frequency score associations with local efficiency in the PE-group. The estimates, uncorrected p-values and Bonferroni corrected p-values are provided per region of interest. L=left, R=right.

| Region of interest | estimate | p-value | p-value corrected |
| --- | --- | --- | --- |
| Precentral L | -0.0015 | 0.16 | 1 |
| Precentral R | 0.0006 | 0.55 | 1 |
| Frontal Superior L | 0.0004 | 0.81 | 1 |
| Frontal Superior R | 0.0013 | 0.19 | 1 |
| Frontal Superior Orbital L | 0.0001 | 0.98 | 1 |
| Frontal Superior Orbital R | 0.0012 | 0.50 | 1 |
| Frontal Middle L | -0.0014 | 0.27 | 1 |
| Frontal Middle R | -0.0010 | 0.49 | 1 |
| Frontal Middle Orbital L | 0.0016 | 0.39 | 1 |
| Frontal Middle Orbital R | 0.0018 | 0.17 | 1 |
| Frontal Inferior Oper L | -0.0027 | 0.43 | 1 |
| Frontal Inferior Oper R | -0.0036 | 0.00 | 0.18 |
| Frontal Inferior Pars Triangularis L | -0.0038 | 0.08 | 1 |
| Frontal Inferior Pars Triangularis R | -0.0043 | 0.03 | 1 |
| Frontal Inferior Orbital L | -0.0002 | 0.88 | 1 |
| Frontal Inferior Orbital R | 0.0027 | 0.10 | 1 |
| Rolandic Oper L | -0.0028 | 0.05 | 1 |
| Rolandic Oper R | -0.0046 | 0.09 | 1 |
| Supplemenatry Motor Area L | -0.0002 | 0.90 | 1 |
| Supplemenatry Motor Area R | 0.0005 | 0.78 | 1 |
| Olfactory L | 0.0001 | 0.97 | 1 |
| Olfactory R | 0.0009 | 0.47 | 1 |
| Frontal Superior Medial L | 0.0010 | 0.50 | 1 |
| Frontal Superior Medial R | 0.0003 | 0.81 | 1 |
| Frontal Medial Orbital L | -0.0027 | 0.10 | 1 |
| Frontal Medial Orbital R | -0.0006 | 0.62 | 1 |
| Rectus L | -0.0007 | 0.72 | 1 |
| Rectus R | 0.0006 | 0.62 | 1 |
| Insula L | -0.0028 | 0.01 | 0.63 |
| Insula R | 0.0002 | 0.85 | 1 |
| Cingulum Anterios L | -0.0008 | 0.53 | 1 |
| Cingulum Anterios R | -0.0002 | 0.91 | 1 |
| Cingulum Middle L | -0.0007 | 0.70 | 1 |
| Cingulum Middle R | -0.0008 | 0.44 | 1 |
| Cingulum Posterior L | -0.0007 | 0.66 | 1 |
| Cingulum Posterior R | -0.0008 | 0.69 | 1 |
| Hippocampus L | 0.0002 | 0.92 | 1 |
| Hippocampus R | 0.0006 | 0.85 | 1 |
| ParaHippocampal L | 0.0002 | 0.89 | 1 |
| ParaHippocampal R | 0.0019 | 0.62 | 1 |
| Amygdala L | 0.0004 | 0.76 | 1 |
| Amygdala R | 0.0020 | 0.24 | 1 |
| Calcarine L | 0.0021 | 0.03 | 1 |
| Calcarine R | 0.0005 | 0.80 | 1 |
| Cuneus L | 0.0025 | 0.03 | 1 |
| Cuneus R | 0.0031 | 0.00 | 0.40 |
| Lingual L | 0.0023 | 0.03 | 1 |
| Lingual R | 0.0023 | 0.22 | 1 |
| Occipital Superior L | 0.0013 | 0.18 | 1 |
| Occipital Superior R | 0.0008 | 0.65 | 1 |
| Occipital Middle L | 0.0024 | 0.14 | 1 |
| Occipital Middle R | 0.0005 | 0.70 | 1 |
| Occipital Inferior L | 0.0004 | 0.79 | 1 |
| Occipital Inferior R | -0.0006 | 0.71 | 1 |
| Fusiform L | 0.0005 | 0.67 | 1 |
| Fusiform R | 0.0028 | 0.41 | 1 |
| Postcentral L | -0.0028 | 0.04 | 1 |
| Postcentral R | 0.0002 | 0.91 | 1 |
| Parietal Superior L | -0.0014 | 0.52 | 1 |
| Parietal Superior R | 0.0008 | 0.57 | 1 |
| Parietal Inferior L | -0.0008 | 0.63 | 1 |
| Parietal Inferior R | -0.0013 | 0.34 | 1 |
| SupraMarginal L | -0.0023 | 0.46 | 1 |
| SupraMarginal R | -0.0002 | 0.93 | 1 |
| Angular L | -0.0023 | 0.20 | 1 |
| Angular R | -0.0025 | 0.49 | 1 |
| Precuneus L | -0.0005 | 0.72 | 1 |
| Precuneus R | 0.0003 | 0.84 | 1 |
| Paracentral Lobule L | -0.0004 | 0.75 | 1 |
| Paracentral Lobule R | 0.0006 | 0.73 | 1 |
| Caudate L | -0.0001 | 0.95 | 1 |
| Caudate R | 0.0011 | 0.56 | 1 |
| Putamen L | -0.0014 | 0.25 | 1 |
| Putamen R | 0.0013 | 0.22 | 1 |
| Pallidum L | -0.0006 | 0.62 | 1 |
| Pallidum R | -0.0008 | 0.62 | 1 |
| Thalamus L | -0.0005 | 0.74 | 1 |
| Thalamus R | -0.0003 | 0.84 | 1 |
| Heschl L | 0.0005 | 0.68 | 1 |
| Heschl R | -0.0054 | 0.01 | 0.70 |
| Temporal Superior L | -0.0025 | 0.19 | 1 |
| Temporal Superior R | -0.0024 | 0.19 | 1 |
| TemporalPole Superior L | -0.0004 | 0.90 | 1 |
| TemporalPole Superior R | 0.0026 | 0.15 | 1 |
| Temporal Middle L | -0.0011 | 0.76 | 1 |
| Temporal Middle R | 0.0007 | 0.52 | 1 |
| TemporalPole Middle L | 0.0015 | 0.35 | 1 |
| TemporalPole Middle R | 0.0032 | 0.00 | 0.14 |
| Temporal Inferior L | 0.0001 | 0.96 | 1 |
| Temporal Inferior R | 0.0012 | 0.28 | 1 |

Table 5. CAPE positive symptom frequency score associations with clustering coefficient in the PE-group. The estimates, uncorrected p-values and Bonferroni corrected p-values are provided per region of interest.

| Region of interest | estimate | p-value | p-value corrected |
| --- | --- | --- | --- |
| Precentral L | -0.0010 | 0.39 | 1 |
| Precentral R | 0.0008 | 0.48 | 1 |
| Frontal Superior L | 0.0005 | 0.80 | 1 |
| Frontal Superior R | 0.0012 | 0.26 | 1 |
| Frontal Superior Orbital L | 0.0000 | 0.99 | 1 |
| Frontal Superior Orbital R | 0.0009 | 0.63 | 1 |
| Frontal Middle L | -0.0014 | 0.28 | 1 |
| Frontal Middle R | -0.0005 | 0.75 | 1 |
| Frontal Middle Orbital L | 0.0010 | 0.62 | 1 |
| Frontal Middle Orbital R | 0.0015 | 0.30 | 1 |
| Frontal Inferior Oper L | -0.0012 | 0.73 | 1 |
| Frontal Inferior Oper R | -0.0028 | 0.03 | 1 |
| Frontal Inferior Pars Triangularis L | -0.0035 | 0.12 | 1 |
| Frontal Inferior Pars Triangularis R | -0.0038 | 0.07 | 1 |
| Frontal Inferior Orbital L | -0.0005 | 0.71 | 1 |
| Frontal Inferior Orbital R | 0.0023 | 0.17 | 1 |
| Rolandic Oper L | -0.0017 | 0.24 | 1 |
| Rolandic Oper R | -0.0034 | 0.22 | 1 |
| Supplemenatry Motor Area L | 0.0000 | 0.99 | 1 |
| Supplemenatry Motor Area R | 0.0002 | 0.92 | 1 |
| Olfactory L | 0.0001 | 0.98 | 1 |
| Olfactory R | 0.0009 | 0.53 | 1 |
| Frontal Superior Medial L | 0.0009 | 0.57 | 1 |
| Frontal Superior Medial R | 0.0002 | 0.90 | 1 |
| Frontal Medial Orbital L | -0.0034 | 0.05 | 1 |
| Frontal Medial Orbital R | -0.0011 | 0.39 | 1 |
| Rectus L | -0.0007 | 0.73 | 1 |
| Rectus R | 0.0002 | 0.86 | 1 |
| Insula L | -0.0027 | 0.02 | 1 |
| Insula R | -0.0001 | 0.92 | 1 |
| Cingulum Anterios L | -0.0009 | 0.49 | 1 |
| Cingulum Anterios R | -0.0007 | 0.68 | 1 |
| Cingulum Middle L | -0.0014 | 0.45 | 1 |
| Cingulum Middle R | -0.0013 | 0.28 | 1 |
| Cingulum Posterior L | -0.0012 | 0.48 | 1 |
| Cingulum Posterior R | -0.0008 | 0.69 | 1 |
| Hippocampus L | 0.0000 | 0.99 | 1 |
| Hippocampus R | 0.0005 | 0.89 | 1 |
| ParaHippocampal L | 0.0003 | 0.88 | 1 |
| ParaHippocampal R | 0.0021 | 0.59 | 1 |
| Amygdala L | 0.0004 | 0.77 | 1 |
| Amygdala R | 0.0016 | 0.35 | 1 |
| Calcarine L | 0.0021 | 0.05 | 1 |
| Calcarine R | 0.0009 | 0.69 | 1 |
| Cuneus L | 0.0025 | 0.04 | 1 |
| Cuneus R | 0.0041 | 0.00 | 0.06 |
| Lingual L | 0.0022 | 0.05 | 1 |
| Lingual R | 0.0024 | 0.21 | 1 |
| Occipital Superior L | 0.0012 | 0.24 | 1 |
| Occipital Superior R | 0.0007 | 0.73 | 1 |
| Occipital Middle L | 0.0021 | 0.21 | 1 |
| Occipital Middle R | 0.0004 | 0.78 | 1 |
| Occipital Inferior L | 0.0010 | 0.54 | 1 |
| Occipital Inferior R | 0.0001 | 0.93 | 1 |
| Fusiform L | 0.0009 | 0.51 | 1 |
| Fusiform R | 0.0029 | 0.40 | 1 |
| Postcentral L | -0.0022 | 0.12 | 1 |
| Postcentral R | 0.0002 | 0.93 | 1 |
| Parietal Superior L | -0.0009 | 0.69 | 1 |
| Parietal Superior R | 0.0008 | 0.62 | 1 |
| Parietal Inferior L | -0.0003 | 0.88 | 1 |
| Parietal Inferior R | -0.0014 | 0.32 | 1 |
| SupraMarginal L | -0.0018 | 0.58 | 1 |
| SupraMarginal R | 0.0005 | 0.80 | 1 |
| Angular L | -0.0022 | 0.23 | 1 |
| Angular R | -0.0029 | 0.43 | 1 |
| Precuneus L | -0.0005 | 0.73 | 1 |
| Precuneus R | 0.0005 | 0.79 | 1 |
| Paracentral Lobule L | 0.0000 | 0.98 | 1 |
| Paracentral Lobule R | 0.0008 | 0.62 | 1 |
| Caudate L | -0.0002 | 0.90 | 1 |
| Caudate R | 0.0008 | 0.66 | 1 |
| Putamen L | -0.0014 | 0.28 | 1 |
| Putamen R | 0.0010 | 0.37 | 1 |
| Pallidum L | -0.0007 | 0.63 | 1 |
| Pallidum R | -0.0010 | 0.56 | 1 |
| Thalamus L | -0.0006 | 0.70 | 1 |
| Thalamus R | -0.0006 | 0.72 | 1 |
| Heschl L | 0.0008 | 0.53 | 1 |
| Heschl R | -0.0032 | 0.13 | 1 |
| Temporal Superior L | -0.0024 | 0.21 | 1 |
| Temporal Superior R | -0.0023 | 0.23 | 1 |
| TemporalPole Superior L | -0.0005 | 0.89 | 1 |
| TemporalPole Superior R | 0.0026 | 0.17 | 1 |
| Temporal Middle L | -0.0009 | 0.81 | 1 |
| Temporal Middle R | 0.0004 | 0.74 | 1 |
| TemporalPole Middle L | 0.0013 | 0.46 | 1 |
| TemporalPole Middle R | 0.0028 | 0.01 | 0.95 |
| Temporal Inferior L | 0.0002 | 0.89 | 1 |
| Temporal Inferior R | 0.0010 | 0.40 | 1 |

Table 6. CAPE positive symptom distress score associations with local efficiency in the PE-group. The estimates, uncorrected p-values and Bonferroni corrected p-values are provided per region of interest.

| Region of interest | estimate | p-value | p-value corrected |
| --- | --- | --- | --- |
| Precentral L | -0.0015 | 0.18 | 1 |
| Precentral R | 0.0014 | 0.21 | 1 |
| Frontal Superior L | 0.0011 | 0.57 | 1 |
| Frontal Superior R | 0.0008 | 0.44 | 1 |
| Frontal Superior Orbital L | -0.0005 | 0.79 | 1 |
| Frontal Superior Orbital R | 0.0018 | 0.36 | 1 |
| Frontal Middle L | -0.0011 | 0.39 | 1 |
| Frontal Middle R | -0.0008 | 0.61 | 1 |
| Frontal Middle Orbital L | 0.0015 | 0.45 | 1 |
| Frontal Middle Orbital R | 0.0014 | 0.29 | 1 |
| Frontal Inferior Oper L | -0.0028 | 0.43 | 1 |
| Frontal Inferior Oper R | -0.0028 | 0.02 | 1 |
| Frontal Inferior Pars Triangularis L | -0.0017 | 0.46 | 1 |
| Frontal Inferior Pars Triangularis R | -0.0024 | 0.25 | 1 |
| Frontal Inferior Orbital L | -0.0012 | 0.39 | 1 |
| Frontal Inferior Orbital R | 0.0026 | 0.12 | 1 |
| Rolandic Oper L | -0.0037 | 0.01 | 0.86 |
| Rolandic Oper R | -0.0050 | 0.07 | 1 |
| Supplemenatry Motor Area L | 0.0007 | 0.71 | 1 |
| Supplemenatry Motor Area R | 0.0003 | 0.86 | 1 |
| Olfactory L | 0.0015 | 0.72 | 1 |
| Olfactory R | 0.0017 | 0.22 | 1 |
| Frontal Superior Medial L | 0.0006 | 0.69 | 1 |
| Frontal Superior Medial R | 0.0001 | 0.96 | 1 |
| Frontal Medial Orbital L | -0.0033 | 0.05 | 1 |
| Frontal Medial Orbital R | 0.0007 | 0.53 | 1 |
| Rectus L | -0.0003 | 0.86 | 1 |
| Rectus R | 0.0016 | 0.24 | 1 |
| Insula L | -0.0026 | 0.02 | 1 |
| Insula R | 0.0007 | 0.62 | 1 |
| Cingulum Anterios L | 0.0003 | 0.84 | 1 |
| Cingulum Anterios R | 0.0006 | 0.72 | 1 |
| Cingulum Middle L | 0.0004 | 0.82 | 1 |
| Cingulum Middle R | -0.0005 | 0.66 | 1 |
| Cingulum Posterior L | -0.0004 | 0.80 | 1 |
| Cingulum Posterior R | -0.0002 | 0.94 | 1 |
| Hippocampus L | 0.0008 | 0.69 | 1 |
| Hippocampus R | 0.0008 | 0.83 | 1 |
| ParaHippocampal L | 0.0013 | 0.49 | 1 |
| ParaHippocampal R | 0.0009 | 0.81 | 1 |
| Amygdala L | 0.0010 | 0.44 | 1 |
| Amygdala R | 0.0024 | 0.16 | 1 |
| Calcarine L | 0.0021 | 0.03 | 1 |
| Calcarine R | 0.0014 | 0.54 | 1 |
| Cuneus L | 0.0024 | 0.04 | 1 |
| Cuneus R | 0.0033 | 0.00 | 0.40 |
| Lingual L | 0.0019 | 0.08 | 1 |
| Lingual R | 0.0021 | 0.29 | 1 |
| Occipital Superior L | 0.0010 | 0.33 | 1 |
| Occipital Superior R | 0.0018 | 0.35 | 1 |
| Occipital Middle L | 0.0020 | 0.23 | 1 |
| Occipital Middle R | 0.0021 | 0.13 | 1 |
| Occipital Inferior L | 0.0031 | 0.04 | 1 |
| Occipital Inferior R | -0.0004 | 0.84 | 1 |
| Fusiform L | 0.0010 | 0.46 | 1 |
| Fusiform R | 0.0025 | 0.48 | 1 |
| Postcentral L | -0.0020 | 0.14 | 1 |
| Postcentral R | 0.0007 | 0.75 | 1 |
| Parietal Superior L | -0.0007 | 0.74 | 1 |
| Parietal Superior R | 0.0011 | 0.47 | 1 |
| Parietal Inferior L | -0.0002 | 0.93 | 1 |
| Parietal Inferior R | -0.0002 | 0.89 | 1 |
| SupraMarginal L | -0.0012 | 0.70 | 1 |
| SupraMarginal R | 0.0009 | 0.66 | 1 |
| Angular L | 0.0000 | 0.99 | 1 |
| Angular R | -0.0013 | 0.73 | 1 |
| Precuneus L | -0.0003 | 0.83 | 1 |
| Precuneus R | 0.0004 | 0.82 | 1 |
| Paracentral Lobule L | 0.0007 | 0.58 | 1 |
| Paracentral Lobule R | 0.0014 | 0.40 | 1 |
| Caudate L | 0.0004 | 0.79 | 1 |
| Caudate R | 0.0014 | 0.47 | 1 |
| Putamen L | -0.0008 | 0.51 | 1 |
| Putamen R | 0.0019 | 0.08 | 1 |
| Pallidum L | -0.0006 | 0.66 | 1 |
| Pallidum R | 0.0001 | 0.96 | 1 |
| Thalamus L | -0.0006 | 0.68 | 1 |
| Thalamus R | 0.0001 | 0.95 | 1 |
| Heschl L | 0.0015 | 0.21 | 1 |
| Heschl R | -0.0052 | 0.01 | 1 |
| Temporal Superior L | -0.0020 | 0.29 | 1 |
| Temporal Superior R | -0.0021 | 0.27 | 1 |
| TemporalPole Superior L | -0.0011 | 0.76 | 1 |
| TemporalPole Superior R | 0.0031 | 0.10 | 1 |
| Temporal Middle L | -0.0013 | 0.73 | 1 |
| Temporal Middle R | 0.0008 | 0.50 | 1 |
| TemporalPole Middle L | 0.0014 | 0.41 | 1 |
| TemporalPole Middle R | 0.0040 | 0.00 | 0.01 |
| Temporal Inferior L | -0.0005 | 0.78 | 1 |
| Temporal Inferior R | 0.0015 | 0.18 | 1 |

Table 7. CAPE positive symptom distress score associations with clustering coefficient in the PE-group. The estimates, uncorrected p-values and Bonferroni corrected p-values are provided per region of interest.

| Region of interest | estimate | p-value | p value corrected |
| --- | --- | --- | --- |
| Precentral L | -0.0010 | 0.43 | 1 |
| Precentral R | 0.0016 | 0.18 | 1 |
| Frontal Superior L | 0.0013 | 0.53 | 1 |
| Frontal Superior R | 0.0008 | 0.45 | 1 |
| Frontal Superior Orbital L | -0.0005 | 0.83 | 1 |
| Frontal Superior Orbital R | 0.0018 | 0.38 | 1 |
| Frontal Middle L | -0.0009 | 0.52 | 1 |
| Frontal Middle R | -0.0002 | 0.93 | 1 |
| Frontal Middle Orbital L | 0.0012 | 0.55 | 1 |
| Frontal Middle Orbital R | 0.0014 | 0.33 | 1 |
| Frontal Inferior Oper L | -0.0011 | 0.75 | 1 |
| Frontal Inferior Oper R | -0.0021 | 0.11 | 1 |
| Frontal Inferior Pars Triangularis L | -0.0016 | 0.50 | 1 |
| Frontal Inferior Pars Triangularis R | -0.0019 | 0.38 | 1 |
| Frontal Inferior Orbital L | -0.0013 | 0.41 | 1 |
| Frontal Inferior Orbital R | 0.0026 | 0.14 | 1 |
| Rolandic Oper L | -0.0028 | 0.07 | 1 |
| Rolandic Oper R | -0.0037 | 0.20 | 1 |
| Supplemenatry Motor Area L | 0.0012 | 0.56 | 1 |
| Supplemenatry Motor Area R | 0.0001 | 0.97 | 1 |
| Olfactory L | 0.0015 | 0.71 | 1 |
| Olfactory R | 0.0017 | 0.24 | 1 |
| Frontal Superior Medial L | 0.0009 | 0.59 | 1 |
| Frontal Superior Medial R | 0.0000 | 0.97 | 1 |
| Frontal Medial Orbital L | -0.0040 | 0.02 | 1 |
| Frontal Medial Orbital R | 0.0004 | 0.78 | 1 |
| Rectus L | -0.0003 | 0.89 | 1 |
| Rectus R | 0.0014 | 0.31 | 1 |
| Insula L | -0.0023 | 0.06 | 1 |
| Insula R | 0.0006 | 0.68 | 1 |
| Cingulum Anterios L | 0.0001 | 0.95 | 1 |
| Cingulum Anterios R | 0.0001 | 0.98 | 1 |
| Cingulum Middle L | -0.0002 | 0.92 | 1 |
| Cingulum Middle R | -0.0010 | 0.42 | 1 |
| Cingulum Posterior L | -0.0007 | 0.71 | 1 |
| Cingulum Posterior R | -0.0001 | 0.96 | 1 |
| Hippocampus L | 0.0009 | 0.67 | 1 |
| Hippocampus R | 0.0009 | 0.80 | 1 |
| ParaHippocampal L | 0.0012 | 0.53 | 1 |
| ParaHippocampal R | 0.0014 | 0.73 | 1 |
| Amygdala L | 0.0010 | 0.50 | 1 |
| Amygdala R | 0.0025 | 0.16 | 1 |
| Calcarine L | 0.0024 | 0.03 | 1 |
| Calcarine R | 0.0020 | 0.39 | 1 |
| Cuneus L | 0.0028 | 0.03 | 1 |
| Cuneus R | 0.0043 | 0.00 | 0.05 |
| Lingual L | 0.0022 | 0.06 | 1 |
| Lingual R | 0.0023 | 0.27 | 1 |
| Occipital Superior L | 0.0010 | 0.36 | 1 |
| Occipital Superior R | 0.0020 | 0.31 | 1 |
| Occipital Middle L | 0.0020 | 0.25 | 1 |
| Occipital Middle R | 0.0024 | 0.10 | 1 |
| Occipital Inferior L | 0.0042 | 0.01 | 0.75 |
| Occipital Inferior R | 0.0009 | 0.62 | 1 |
| Fusiform L | 0.0015 | 0.30 | 1 |
| Fusiform R | 0.0025 | 0.49 | 1 |
| Postcentral L | -0.0016 | 0.28 | 1 |
| Postcentral R | 0.0006 | 0.78 | 1 |
| Parietal Superior L | -0.0003 | 0.91 | 1 |
| Parietal Superior R | 0.0012 | 0.43 | 1 |
| Parietal Inferior L | 0.0003 | 0.85 | 1 |
| Parietal Inferior R | 0.0001 | 0.96 | 1 |
| SupraMarginal L | -0.0007 | 0.85 | 1 |
| SupraMarginal R | 0.0014 | 0.50 | 1 |
| Angular L | -0.0002 | 0.91 | 1 |
| Angular R | -0.0013 | 0.74 | 1 |
| Precuneus L | -0.0002 | 0.92 | 1 |
| Precuneus R | 0.0006 | 0.72 | 1 |
| Paracentral Lobule L | 0.0008 | 0.55 | 1 |
| Paracentral Lobule R | 0.0014 | 0.41 | 1 |
| Caudate L | 0.0004 | 0.78 | 1 |
| Caudate R | 0.0014 | 0.49 | 1 |
| Putamen L | -0.0007 | 0.59 | 1 |
| Putamen R | 0.0019 | 0.11 | 1 |
| Pallidum L | -0.0005 | 0.74 | 1 |
| Pallidum R | 0.0002 | 0.89 | 1 |
| Thalamus L | -0.0005 | 0.72 | 1 |
| Thalamus R | 0.0001 | 0.94 | 1 |
| Heschl L | 0.0015 | 0.23 | 1 |
| Heschl R | -0.0031 | 0.16 | 1 |
| Temporal Superior L | -0.0019 | 0.35 | 1 |
| Temporal Superior R | -0.0018 | 0.36 | 1 |
| TemporalPole Superior L | -0.0008 | 0.84 | 1 |
| TemporalPole Superior R | 0.0034 | 0.08 | 1 |
| Temporal Middle L | -0.0009 | 0.82 | 1 |
| Temporal Middle R | 0.0008 | 0.54 | 1 |
| TemporalPole Middle L | 0.0015 | 0.41 | 1 |
| TemporalPole Middle R | 0.0039 | 0.00 | 0.05 |
| Temporal Inferior L | -0.0001 | 0.96 | 1 |
| Temporal Inferior R | 0.0017 | 0.16 | 1 |

Table 8. Association between attenuated symptoms and network connectivity measures within the PE group. Analyses are corrected for total MADRS score.

|  | global efficiency | | local efficiency | | clustering coefficient | |
| --- | --- | --- | --- | --- | --- | --- |
|  | estimate | p-value | χ^2^ | p-value | χ^2^ | p-value |
| CAPE positive frequency score | 0.00020 | 0.99 | 149.91 | p<0.0001 | 127.02 | 0.005 |
| CAPE positive distress score | 0.00038 | 0.99 | 138.26 | 0.0006 | 128.44 | 0.005 |
| CAPE negative frequency score | 0.00063 | 0.78 | 120.05 | 0.02 | 107.61 | 0.10 |
| CAPE negative distress score | 0.00031 | 0.97 | 97.66 | 0.27 | 86.93 | 0.57 |
| CAPE depressive frequency score | 0.00041 | 0.99 | 93.17 | 0.39 | 80.27 | 0.76 |
| CAPE depressive distress score | 0.00017 | 0.99 | 88.39 | 0.53 | 83.91 | .0.66 |
| CAPE total frequency score | 0.00028 | 0.92 | 119.45 | 0.02 | 100.95 | 0.20 |
| CAPE total distress score | 0.00015 | 0.99 | 103.21 | 0.16 | 90.66 | 0.46 |
| Daily life ESM PE- score | -0.0076 | 0.99 | 78.03 | 0.81 | 72.66 | 0.91 |

MADRS; Montgomery–Åsberg Depression Rating Scale, ESM; Experience Sampling Method. χ^2^, estimates and p-values are derived from multilevel random regression analyses (local efficiency and clustering coefficient) and regression for global efficiency.
